# Psychiatric and criminological profile of female inpatients in Tunisia

**DOI:** 10.1192/j.eurpsy.2023.1871

**Published:** 2023-07-19

**Authors:** R. Felhi, G. Hamdi, S. Chebli, R. Ridha

**Affiliations:** Razi hospital, Manouba

## Abstract

**Introduction:**

Studies of criminality among women are relatively poor because of its lower rate compared to men and its frequent association with mental illness.

**Objectives:**

The aim of this study is to determine the characteristics of female offenders referred for forensic psychiatric examination.

**Methods:**

We studied the medical files of all the offenders referred to the forensic psychiatry unit in the Razi hospital for an examination between January 2010 and October 2020.

**Results:**

The number of people who have undergone a forensic psychiatric examination was 256. The offenders were female in 4.29% (11) of the cases. Their average age was 35 years with a range of 17-73 years. They were mostly single (54.5%) with no education (54%). Fifty four percent of the studied population were unemployed and 63% of them lived with their families.

One third of the studied population had a neurological history. Family history of psychiatric disorder was found in one case and only two had suicide attempts history.

Two women had personality disorder: a borderline and a histrionic personality disorder. An average number of one hospitalization was found in this group.

A criminal record was found in 47% of the cases with an average number of two offenses per person. The mean age of the first offense was 24 years old. The main crimes were: homicide (18.2%) and theft (18.2%).

Three offenders were found of intellectual disability, two of them personality disorders, one of schizophrenia, one of dementia and one of depression. No psychiatric disorder was found in the rest of the cases.

No drug abuse was found in this population.

**Conclusions:**

The criminal and psychiatric profile of female inpatients differs from their male counterparts, which has important involvements in case management and validates the need of further searches in this field.

**Disclosure of Interest:**

None Declared

